# Perovskite Nanocrystals, Quantum Dots, and Two-Dimensional Structures: Synthesis, Optoelectronics, Quantum Technologies, and Biomedical Imaging

**DOI:** 10.3390/nano16010030

**Published:** 2025-12-25

**Authors:** Kamran Ullah, Anwar Ul Haq, Sergii Golovynskyi, Tarak Hidouri, Junle Qu, Iuliia Golovynska

**Affiliations:** 1 College of Physics and Optoelectronic Engineering, Shenzhen University, Shenzhen 518060, China; kamranullah2025@email.szu.edu.cn (K.U.); 2451233008@mails.szu.edu.cn (A.U.H.); jlqu@szu.edu.cn (J.Q.); iuliia@szu.edu.cn (I.G.); 2 Department of Mathematical, Physical and Computer Sciences, University of Parma, 43124 Parma, Italy; tarek.hidouri@unipr.it

**Keywords:** perovskite, quantum dot, 2D structure, photoluminescence, solar cell, photodetector, light-emitting diode

## Abstract

Perovskite crystals, nanocrystals, quantum dots (QDs), and two-dimensional (2D) materials are at the forefront of optoelectronics and quantum optics, offering groundbreaking potential for a wide range of applications, including photovoltaics, light-emitting devices, and quantum information technologies. Perovskite materials, with their remarkable, tunable bandgaps, high absorption coefficients, and efficient charge transport, have revolutionized the field of light-emitting diodes, photodetectors, and solar cells. QDs, owing to their size-dependent quantum confinement and high photoluminescence quantum yields, are crucial for applications in display technologies, imaging, and quantum computing. The synthesis of QDs from perovskite-based materials yields a significant enhancement in the performance of optoelectronics devices. Furthermore, 2D perovskites have recently exhibited extraordinary carrier mobility, strong light–matter interactions, and mechanical flexibility, making them highly attractive for next-generation optoelectronic applications. Additionally, this review discusses the synergistic potential of hybrid material architectures, where perovskite crystals, QDs, and 2D materials are combined to enhance optoelectronic performance and their role in quantum optics. By analyzing the effects of material structure, surface modifications, and fabrication techniques, this review provides a valuable resource for harnessing the transformative potential of these advanced materials in modern optoelectronic applications.

## 1. Introduction

Metal-halide perovskite (MHP) semiconductors have emerged as a transformative class of materials in the fields of optoelectronics and quantum optics. Their general chemical formula is ABX_3_, where A is a monovalent cation, e.g., methylammonium (MA^+^, CH_3_NH_3_^+^), formamidinium (FA^+^, HC(NH_2_)^2+^), or cesium (Cs^+^). In this manuscript, the names of the corresponding organic species, including MA, FA, BA (butylammonium), and GA (guanidinium) are abbreviated, but the addition sign is explicitly stated when the cationic form is discussed in the context of the perovskite lattice. They possess exceptional properties, such as high absorption coefficients, tunable bandgaps, and efficient charge transport [1,2,3,4]. These attributes make perovskites suitable for a wide range of applications, including perovskite solar cells (PSCs) [5], light-emitting diodes (PeLEDs) [6], photodetectors (PDs) [7], lasers [8], and other optoelectronic devices. In particular, recent advancements in MHP materials have captivated the interest of researchers owing to their intriguing properties and exceptional performances in light-matter interactions, particularly in quantum optic devices [9]. Except for perovskite crystals and perovskite thin films (PTFs), perovskite materials can be divided into perovskite nanocrystals (PNCs), perovskite quantum dots (PQDs), and two-dimensional perovskite structures (2DPs). PNCs are nanoscale particles with sizes below hundreds of nanometers but larger than the quantum confinement regime (i.e., significantly larger than the exciton Bohr diameter) [10]. PNCs can also have tunable optical and electrical properties due to their chemical composition. PNCs tend to favor tetragonal or orthorhombic symmetries at room temperature (RT) while transitioning to a more stable cubic phase as the temperature increases. PNCs exhibit high photoluminescence quantum yields (PLQYs), often above 90%, and absorb broadly across the visible (Vis) range. CsPbBr_3_ is one of the most efficient and stable PNCs. PNCs are widely used in PeLEDs, lasers, PSCs, and PDs and are being explored for quantum light sources due to their tunable bright emission and potential for single-photon generation. With low-cost fabrication and excellent performance, they offer strong potential for next-generation optoelectronic devices and quantum technologies [11].

The perovskite bulk phase is unstable; to overcome this notable phase instability, perovskite has been processed into QDs. The PQDs are semiconductor NCs that are characterized by peculiar optoelectronic characteristics arising from the nanoscale size of the particles, which results in an intense quantum confinement effect [12]. When the size of these semiconductor PNCs is reduced to less than approximately 10 nm, the electronic bandgap and photoelectrical characteristics of these nanoparticles (NPs) are extensively tuned, resulting in improved structural stability. Simultaneously, such downsizing also reveals desirable attributes, including wide spectral tunability and outstanding PL performances [13,14]. PQDs may crystallize in cubic, tetragonal, or orthorhombic phases, and the phase transition depends strongly on temperature and composition. They have superb PLQYs, which can be up to 90% under optimum conditions. They can be tuned to any part of the spectrum by virtue of their broad absorption band, spanning from the ultraviolet (UV) to the Vis range. CsPbBr_3_ is one of the most effective PQDs because it is stable and emits intensely. It has applications in high-efficiency displays, SCs, PDs, and bioimaging [15].

In a generalized view of QDs, it is educative to contrast lead-halide PQDs with multinary chalcogenide QDs (group-11/InS(Se), e.g., CuInS_2_, AgInS_2_, CuInSe_2_, etc.) and quaternary systems. These multinary QDs typically have crystal structures of chalcopyrite/wurtzite (ternaries) or kesterite/stannite (quaternaries) instead of perovskite lattice. They have optical emission that tends to be broader and longer-lived, often dominated by recombination via defect and donor–acceptor pairs, which is extremely stoichiometry-sensitive and reactivity-sensitive to cations during synthesis. Comparatively, however, lead-halide PQDs are characterized by relatively narrower band-edge emission and high-speed composition tuning through halide exchange and have different issues associated with the ionic softness, ion movement, and environmental stability [16].

Surface chemistry and passivation/core–shell approaches, however, are definitive in both families in suppressing nonradiative pathways and enhancing photostability, a similarity of synthesis-structure-property theme in various classes of crystalline QDs. To go into greater detail of multinary chalcogenide QDs, the reader is directed to a specific review [17].

Two-dimensional materials are a class of crystalline layered materials with a thickness of one or a few atomic layers and a lateral size much larger than the thickness. 2DPs possess unique physical and chemical properties arising from the quantum confinement effect [18]. For example, when the dimensionality of perovskites is reduced to the atomically thin limit, many of their unique characteristics emerge, accompanied by significantly enhanced stability in ambient conditions and a substantial broadening of their potential application domains [18]. 2DPs offer excellent resilience to moisture, heat, and light relative to their 3D counterparts. Tunable bandgap, high PLQY, and efficient charge transport enable their potential in PeLEDs, high-efficiency PSCs, and PDs [19]. Moreover, 2DPs exhibit exceptional excitonic properties, including considerable binding energies that facilitate efficient light emission at ordinary temperatures [20,21].

**Figure 1:**
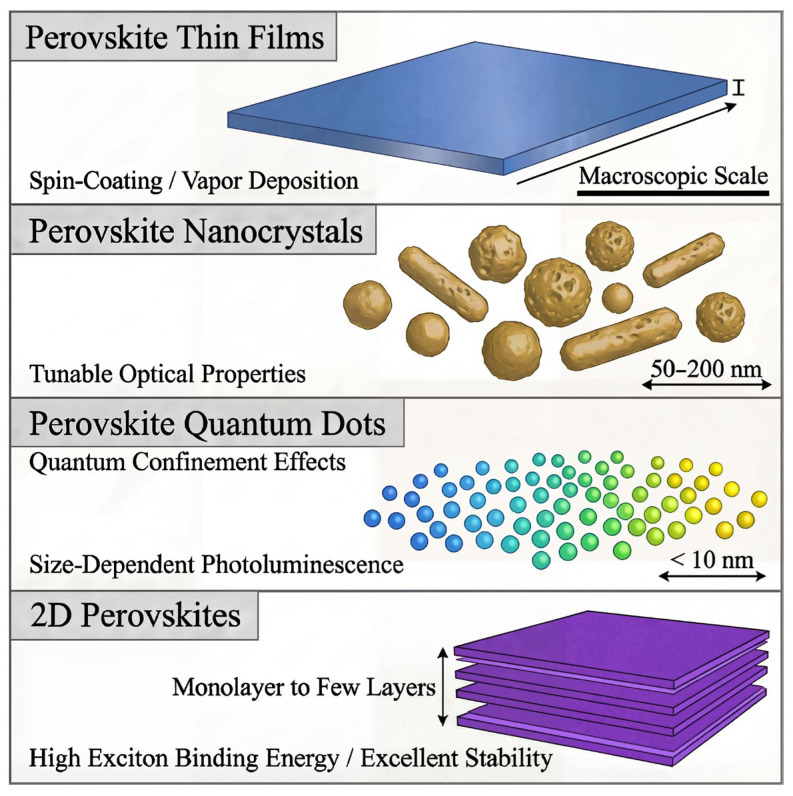
The size and structure of PTFs, PNCs, PQDs, and 2DPs.

Perovskite materials, including thin films, NCs, QDs, and 2D structures, offer diverse optical properties that can be tuned for a wide range of applications. Perovskite thin films, typically fabricated via spin-coating or vapor deposition, exhibit macroscopic scale properties, while NCs and QDs allow for size dependent optical properties, such as tunable PL and quantum confinement effects. 2DPs, consisting of monolayer to few-layer structures, stand out for their high exciton binding energy and exceptional stability as depicted in Figure 1, making them promising for optoelectronic applications. Their low cost and simple solution-based synthesis make them ideal for large-scale applications. Despite the progress, challenges like toxicity (due to Pb) and long-term durability persist. Researchers are actively working on Pb-free alternatives and encapsulation strategies; with continued innovation, PQDs hold great promise for next-generation optoelectronic technologies.

This review presents a comprehensive overview of recent progress in the development and optoelectronic applications of perovskite crystals, PNCs, PQDs, and 2DPs, focusing on the development of each type of structure. First, we introduce their crystal structures and fundamental optical and electronic properties. Next, we examine various synthesis strategies employed and discuss both their advantages and limitations. The practical role of these materials in devices such as PeLEDs, lasers, PSCs, and PDs is analyzed in detail. By providing a comprehensive overview of the current progress in perovskite research, this review aims to offer meaningful insights and inspire innovative approaches for future research and the real-world implementation of these promising materials.

## 2. Perovskite Structures

**Figure 2:**
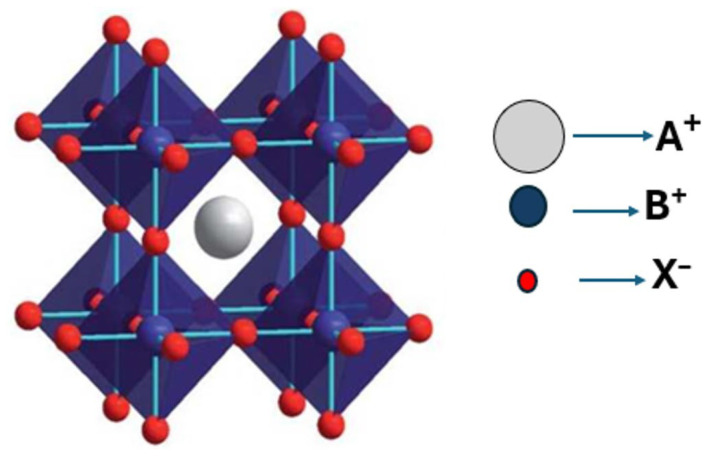
Crystal structure of a halogen-based perovskite within a unit cell. Adopted with permission from reference [23]. Copyright 2018, *Crystal Growth and Design*.

Perovskite structures refer to a broad class of materials that share a similar crystal structure to the mineral perovskite (CaTiO_3_) structure. They follow the general formula ABX_3_, where A and B are cations of different sizes, and X is an anion. The A-site cation occupies the corners, and the B-site cation occupies the body center of the unit cell (Figure 2). In the structural framework crystal, the B-site cation is coordinated octahedrally by six X anions, resulting in the formation of BX_6_ octahedra, which are interconnected at their corners to create a three-dimensional (3D) network. Meanwhile, the A-site cation occupies the 12-coordinated cubo-octahedral voids within this framework, as shown in Figure 2. Herein, the A-site can be occupied by either organic cations, such as methylammonium (MA^+^, CH_3_NH_3_^+^) and formamidinium (FA^+^, HN=CHNH_2_^+^), or by inorganic ones (Cs^+^, Rb^+^, and K^+^), while the B-site is typically composed of divalent metal cations, such as lead (Pb^2+^) or tin (Sn^2+^), and the X-site is occupied by halide anions, including iodide (I^−^), bromide (Br^−^), or chloride (Cl^−^) [22]. To explain the stability of the ABX_3_ perovskite structure, the concept of the Goldschmidt tolerance factor (*t_f_*) is proposed:

(1) $$t_{f}=\frac{\left(R_{A}+R_{X}\right)}{\sqrt{2}(R_{B}+R_{X})}$$

where *R_A_*, *R_B_*, and *R_X_* are the effective radii of A, B, and X, respectively [23].

Perovskite crystals exhibit a stable cubic geometry when the *t_f_* lies between 0.9 and 1.0, as shown in Table 1. The cubic structure predominates at higher temperatures, whereas the tetragonal and orthorhombic phases are more stable at RT. When *t_f_* decreases below 0.9, the structure’s symmetry decreases, yielding distorted frameworks such as tetragonal, orthorhombic, or rhombohedral phases. These distortions are commonly attributed to the differences in ionic radii, such as smaller A-site or larger B-site cations. For *t_f_* values exceeding 1.0, the crystal structure may adopt a hexagonal or tetrahedral configuration, attributed to the larger A-site cation. Thus, the perovskite structure maintains structural stability predominantly when the *t_f_* value ranges between 0.7 and 1.0. The *t_f_* serves as a fundamental guideline for structural stability and related material properties. However, *t_f_* is not the only key factor to comprehensively assess the structural stability of perovskites because the non-geometric factors, including chemical stability and bond valence, also influence the perovskite crystal structure [23]. Independent of the crystal structure and composition, perovskite materials can be categorized into crystals, films, PNCs, PQDs, and 2DPs. This is particularly described below.

### 2.1. Perovskite Crystals and Thin Films

**Figure 3:**
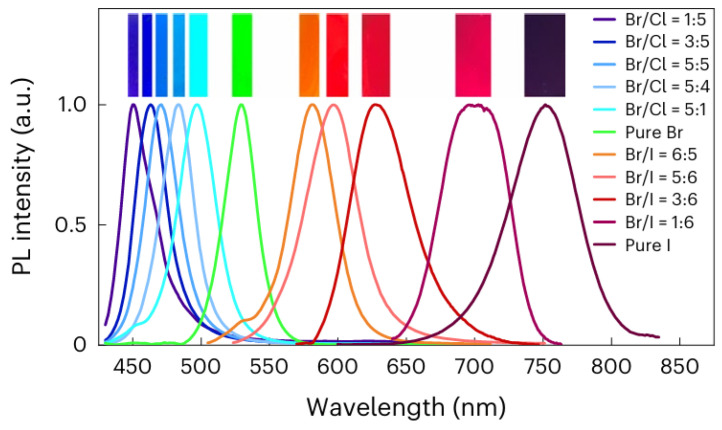
Normalized PL spectra with insets demonstrating the corresponding fluorescent photographs of MAPb(Cl_1−x/_Br_x_)_3_, MAPbBr_3_, MAPb(Br_x_/I_1−x_)_3_, and MAPbI_3_ PTFs [25]. Copyright 2023, *Nature Photonics*.

Perovskite crystals and PTFs, with the general formula of ABX_3_, emerged as a transformative class of materials in optoelectronics due to their exceptional properties and low-cost fabrication. These materials crystallize in cubic, tetragonal, or orthorhombic structures, exhibiting high absorption coefficients (greater than 10^5^ cm^−1^), long charge carrier diffusion lengths (greater than 1 µm), and tunable bandgaps (1.2–3.0 eV) via halide substitution [25,26,27]. Additionally, they demonstrate high PLQY (>90%) in optimized films, making them ideal for PeLED applications. PTFs, typically deposited using solution-based methods such as spin coating or vapor phase deposition techniques, have enabled the remarkable progress in photovoltaic efficiency, with PSCs now exceeding a 26% power conversion efficiency (PCE), rivaling traditional silicon-based technologies [28]. Significant progress was made through innovations in material composition (e.g., mixed cations such as Cs^+^/FA^+^/MA^+^ for enhanced stability and device performance), interfacial passivation (using molecular additives to suppress defects), and fabrication techniques [29]. Additionally, by substituting the halide elements within the perovskite and formulating ink with varying halide ratios, emitters covering the entire Vis spectrum also enhance their efficiency, as shown in Figure 3 [29]. A notable example is the triple cation Cs/FA/MA, Pb(I/Br)_3_ PSCs, which showed 25.7% PCE and retained 80% efficiency after one thousand hours of operation, highlighting improvements in both performance and durability.

Beyond photovoltaics, PTFs are widely explored in PeLEDs, where green-emitting devices have achieved an external quantum efficiency (EQE) exceeding 20% [30], as well as in PDs and lasers due to their high gain and fast response times (<1 ns) [31]. Despite these breakthroughs, several challenges persist, including environmental degradation under moisture, light, and heat; concerns about Pb toxicity; and difficulties in maintaining uniformity during large-scale production. To address these, current research is focused on developing Pb-free alternatives (such as Sn- or Bi-based perovskites), advanced encapsulation methods, and flexible substrate integration for wearable and portable applications. With continued optimization, perovskite crystals and PTFs hold immense potential for commercialization, promising to transform solar energy harvesting, display technologies, and next-generation optoelectronic devices. Their unique combination of tunable optoelectronic properties, cost-effectiveness, and high performance makes them at the forefront of modern materials science and renewable energy innovation [32].

### 2.2. Perovskite Nanocrystals

**Figure 4:**
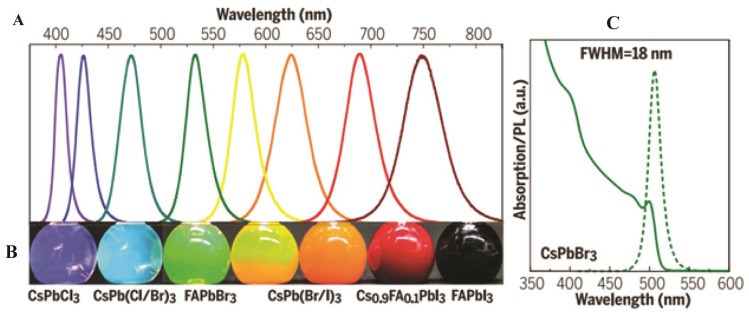
Perovskite-based NCs: (**A**) Survey of PL spectra. (**B**) Photographs under mixed daylight and UV excitations of composition-tuned APbX_3_ PNCs. (**C**) Absorption and PL spectra of CsPbBr_3_ PNCs showing quantum size effect and three well-resolved transitions [11]. Copyright 2017, *Science*.

First reported in 2015, PNCs have rapidly gained attention due to their exceptional PL properties and solution processability [11,14]. These structures offer quantum size effects, enhanced surface-to-volume ratios, and defect-tolerant electronic structures, making them a leading research area in nanomaterials. The most extensively studied PNCs are CsPbX_3_ compounds, including CsPbBr_3_, CsPbI_3_, and CsPbCl_3_, as well as mixed-halide variants. These materials show outstanding optical properties, including narrow emission linewidths (<20 nm), high PLQY (above 95%), and tunable emission wavelength across the Vis and NIR regions (Figure 4A) by changing the composition or the synthesis method, such as ligand-assisted reprecipitation (LARP), the hot-injection method, and RT synthesis, all of which allow precise control over NC morphology [11,33]. As shown in Figure 4A, the PL spectra of composition-tuned APbX_3_ PNCs highlight their broad spectral tunability, while Figure 4B vividly demonstrates the color variation under mixed daylight and UV excitation. Furthermore, the absorption and PL spectra of colloidal CsPbBr_3_ PNCs depicted in Figure 4C reveal apparent quantum-size effects and well-resolved optical transitions, confirming their size-dependent optical characteristics [11].

The PNCs have tunable bandgaps ranging from 1.5 to 3.1 eV for CsPbX_3_ and exhibit excellent charge carrier mobility (greater than 10 cm^2^/V·s). These characteristics make them ideal for a wide range of applications. In LEDs, green-emitting PNCs have achieved EQE exceeding 20%, setting new benchmarks for display technologies [34]. PNCs also exhibit low-threshold amplified spontaneous emissions, paving the way for tunable nanolasers [35]. In PDs, they enable high-gain devices with rapid response times (less than 10 ns), while their scintillation light yields make them promising for X-ray detection [35,36]. Despite their promising features, challenges such as surface degradation, phase instability, Pb toxicity, and scalable production remain active areas of research. Current research addresses these issues through advanced encapsulation methods, developing Pb-free alternatives (e.g., Sn^2+^, Bi^3+^) and large-scale techniques [37]. With their unparalleled optical properties and versatility, PNCs are poised to transform optoelectronic devices, from ultra-efficient displays to next-generation sensors [35].

### 2.3. Perovskite Quantum Dots

**Figure 5:**
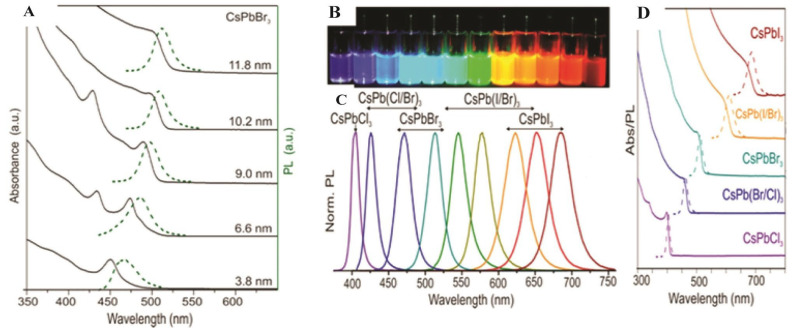
(**A**) Size-tunable absorption and emissions of CsPbBr_3_ QDs. (**B**–**D**) element tunable absorption and emission of CsPbX_3_ QDs. Adopted with permission from reference [14]. Copyright 2015, *American Chemical Society*.

PQDs, a subclass of PNCs, have emerged as cutting-edge materials in the field of optoelectronics due to their peerless optoelectronic properties at the nanoscale. Quantum-confined NPs typically span 2 to 10 nm in size and exhibit unique optoelectronic properties that are strongly influenced by their dimensions and material composition. The nanoscale confinement of PQDs favors their tunable emission across the Vis range [38,39]. This characteristic can be explained in two aspects. A notable aspect is the tunable emission, which is closely linked to the dimensions of PQDs. Kovalenko’s group revealed that modifying the size of CsPbBr_3_ PQDs leads to a noticeable shift in both emission wavelength and absorption peak, as shown in Figure 5A [40,41]. The other aspect is the elemental tunability of emission. The emission spectrum of PQDs can be tuned over a wide range of Vis and near-infrared (NIR) regions by altering the halide anions of CsPbX_3_, where X is Cl^−^, Br^−^, and I^−^, as shown in Figure 5B–D [38]. Moreover, bandgap tunability can also be achieved by adjusting particle size and halide composition. For instance, CsPbX_3_ (X = Cl, Br, I) PQDs show tunable PL across the Vis spectrum (400–780 nm). PQDs offer exceptionally high PLQY, often exceeding 90% for green-emitting CsPbBr_3_, due to efficient radiative recombination and a defect-tolerant electronic structure. Singh et al. [42] synthesized CsPbBr_3_ PQDs and reported blue PL emission at a wavelength of 457 nm, with a narrow full-width at half maximum (FWHM) of 22 nm, and achieved a PLQY of 87% with temperature regulation. These PQDs also possess favorable charge carrier dynamics, characterized by long carrier diffusion lengths, high mobility, low trap density, and short radiative decay times (<100 ps), which are crucial for fast-response optoelectronic devices.

The optoelectronic properties of PQDs depend also on crystal facets and surface termination, which influence the distribution of undercoordinated ions, surface traps, and ligand-binding motifs, in addition to their size and composition. Lead-halide PQDs often take the form of cubic or pseudo-cubic lattice and often present low-index surfaces, being terminated by a dynamic structure of halide and metal ions. Unlike most II-VI QDs, with polar vs. nonpolar facets capable of that strongly directing anisotropic growth and selective adsorption of coordinating ligands (e.g., phosphine oxides like TOPO), the halide PQD surfaces are more ionic and structurally soft, allowing reconstruction and labile binding to nonetheless make (or remake) trap states to depend on the surface chemistry. Hence, it is necessary that the ligand/surface passivation strategies (carboxylates, amines, ammonium halides, zwitterionic ligands, etc.) continue to stabilize individual surface sites, neutralize halide vacancies and undercoordinated Pb^2+^ species, and inhibit nonradiative recombination, maintaining high PLQY and spectral stability [43].

With respect to core–shell ideas, classical epitaxial core–shell growth (as used in II-VI QDs) is typically not easy to realize in the case of PQDs, as their ionic lattices are typically soft and their ion mobility is high, but core–shell-like stabilization is widespread experimentally. These consist of perovskite core–shell structures (e.g., CsPbBr_3_/Cs_4_PbBr_6_) inorganic shells (e.g., SiO_2_), and encapsulation-overcoating designs, which effectively emulate core–shell functions by increasing the chemical strength, decreasing surface-ion flux, and reducing the trap density without decreasing the emission. This supports the development of a structural- and surface-chemistry viewpoint, using PQDs to be explicit and to connect the previous QD body of knowledge to the perovskite field [16].

The optoelectronic application of PQDs is expanding rapidly. In PQD-LEDs (PQLEDs), PQDs have demonstrated high color purity and EQEs exceeding 20% in green and red emissions [44]. In photovoltaics, PQDs are being explored as absorber layers or interfacial components due to their strong absorption coefficients and tunable bandgaps. Their ultrafast charge carrier response and low threshold optical gain also make them suitable for PD and lasers [14].

### 2.4. Two-Dimensional Perovskite Structures

**Figure 6:**
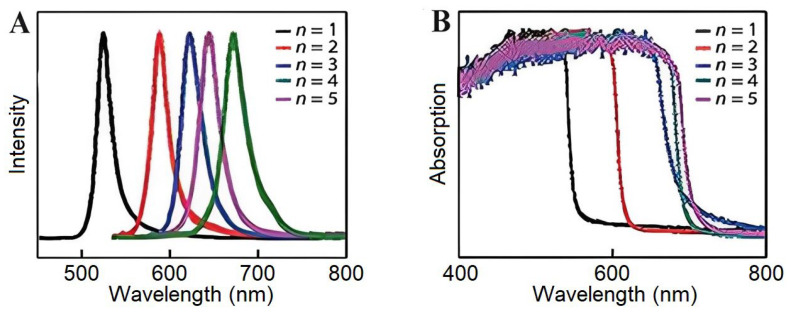
(**A**) Normalized PL spectra of the (BA)_2_(MA)_n−1_Pb_n_I_3n+1_ perovskite for (*n* = 1–5) with a size below 20 nm. (**B**) Absorption spectra of the as-synthesized (BA)_2_(MA)_n−1_Pb_n_I_3n+1_ (*n* = 1–5) plates [51]. Copyright 2019, *Semiconductor*.

2DPs are naturally quantum-well architectures of layered quantum-confined materials with alternating inorganic metal halide octahedral sheets and bulky organic spacer cations. The shape of 2DPs can be controlled by spacer engineering due to their quantum-well superlattice structure, consisting of alternating [PbX_6_]^4−^ inorganic octahedral layers and organic spacer cations [45,46]. Suppressed ion migration and hydrophobicity based on 3D analogs, especially (BA)_2_(GA)Pb_2_I_7_, contribute to their structural stability, with a 94% PLQY retention after 500 bending cycles through octahedral distortion mitigation from pressure [47]. Charge transport in these materials is layer-dependent, and specific structures yield surface carrier diffusion rates of 470 cm^2^/s, approximately 20 times faster than that in the bulk due to widened surface conduction channels [47]. The quantum confinement effect leads to a tunable bandgap (1.73–3.0 eV) due to variations in the *n* value and large exciton binding energies (up to 0.18 eV in the Sn-based variants), resulting from the dielectric mismatch between organic/inorganic layers [48,49]. Compositions formed with optimized formulations, such as (CMA)_2_(FA)Pb_2_I_7_, lead to a record PLQY of 59%, achieved by reducing trap states and suppressing exciton–phonon coupling [50]. We also demonstrate how pentylammonium-modified (BA)_2_PbI_4_ is pinned to a phase and retains directional exciton diffusion over homojunctions at 454 mW/cm^2^ illumination [45,50]. The naturally occurring quantum-well structure and the notable variation in dielectric constants between the organic and inorganic layers lead to novel and significant optoelectronic properties in 2D (BA)_2_(MA)_n−1_Pb_n_I_3n+1_ (1–5 layers) perovskite [51]. Altering the layer number enables a fine-tuning of the quantum confinement effect, resulting in a gradual shift in the emission peak and absorption onset over an extended wavelength range, as shown in Figure 6A,B. These materials are solution-processable and manifest exceptional optoelectronic tunability, while MoS_2_-stabilized devices retain 45% efficiency under 40–50% humidity for 120 h [52].

## 3. Synthesis Approaches

The main approaches of perovskite synthesis are the hot-injection method and RT synthesis. Additionally, several alternative strategies, including the sol–gel method, hydrothermal and solvothermal synthesis, kinetically controlled space confinement (KCSC), solid-state route, and the solution process method, have been developed for the synthesis of perovskite. Through these approaches, the optical behavior of the synthesized perovskites can be tailored by alternating the dopant, precursor ratio, solvent, temperature, and other parameters.

### 3.1. Hot-Injection Method

Schmidt et al. [52] pioneered the synthesis of CH_3_NH_3_PbBr_3_ PNCs through the hot-injection method. The process entails reacting a Pb precursor with oleic acid (OA) and octadecene (ODE), followed by the introduction of an ammonium cation at 80 °C. Medium-chain alkyl ammonium ions effectively stabilize the PNCs, whereas long-chain amines are incompatible within the NC lattice. This protocol was subsequently modified to yield all-inorganic CsPbX_3_ PNCs by injecting a cesium source into a solution of OA, oleylamine (OLA), and PbX_2_ at 140–200 °C, followed by rapid cooling to RT to form CsPbX_3_. The size of the resulting PNCs can be tuned via the injection temperature [53]. The method has gained widespread adoption for producing colloidal PNCs with varied morphologies and compositions. Using this method, Protesescu et al. [53] synthesized FAPbBr_3_ and CsPbBr_3−x_I_x_ QDs with PLQY exceeding 85% and FWHM below 20 nm. PNCs fabricated by this approach are chemically unstable and tend to degrade, as exposure to polar solvents, including water vapor, can trigger phase changes, precipitation, and material degradation, thereby diminishing their optoelectronic efficiency. The improvement in stability and control over the phase transition of CsPbI_3_ PNCs can be achieved by replacing OA with bis-(2,3,4-trimethyepentyl) phosphinic acid, as reported by Wang et al. [54]. The FAPbBr_3_ QDs were prepared by reacting FA-acetate with OA in ODE, while the oleylammonium bromide (OAmBr) was injected at 130 °C. The luminescent wavelength of QDs can be tuned from 470 to 535 nm by altering the temperature [53]. The hot-injection method enables the rapid synthesis of PNCs with uniform size and well-defined morphology. However, its limitations include incompatibility with large-scale synthesis, low reproducibility, and the requirement of stringent inert gas handling.

### 3.2. Room Temperature Synthesis

Although the hot-injection method has enabled the successful synthesis of PNCs with diverse morphologies, it requires highly precise control of reaction temperature. To address this limitation, researchers have explored and validated low-temperature synthesis routes for perovskite nanomaterial [55]. Zhang et al. [55] introduced a synthesis approach known as LARP, which is commonly executed at RT. In order to prepare the MAPbX_3_ QDs, one of the most common methods is the dissolution of the MAX and PbX_2_ in dimethylformamide (DMF) or dimethyl sulfoxide (DMSO). The mixture is then added to OA and OLA to prepare a precursor solution. The precursor is added dropwise to vigorously stirred toluene to produce a dispersion that exhibits intense PL under UV light. The primary role of OA in this reaction is the regulation of crystallization dynamics and crystal grain size, as well as inhibiting the agglomeration of particles and increasing the stability of colloidal QDs. This method facilitated the production of MAPbX_3_ (X = Cl, Br, I) QDs that had adjustable emission wavelengths, PLQY values as high as 70%, and emission peak FWHM between 20 and 50 nm. Song et al. [56] utilized a multiphase synthesis method on MAPbBr_3_ colloidal QDs, in which DMF was the aqueous phase, n-hexane was the oil phase, OA was the surfactant, and tert-butanol was a demulsifier. A suspension was created by adding an appropriate amount of tert-butanol to the DMF solution, which was then centrifuged and subsequently dissolved in toluene. This technique enabled the strict regulation of QD size (2–8 nm) and provided a high PLQY of 80–92%. Wei et al. [57] proposed a simple and rapid method for producing gram-scale amounts of CSPbBr_3_ PNCs at RT. The process involved adding tetrabutylammonium chloride to the Cs and Pb precursor solution under ambient stirring conditions. They used fatty acids as surface-capping counterparts, unlike the conventional methods. They also specifically investigated the impact of fatty acid chain length on the optical behavior of PNCs. The experiment found that short-chain fatty acids have a predilection for reducing the PLQY of PNCs and also increasing the charge carrier mobility, a critical quality in high-performance photovoltaic applications, including SCs and LEDs. RT synthesis offers advantages such as simplicity, energy efficiency, and scalability, but it also comes with several limitations such as slower reaction rates, poor surface passivation, and lower thermal energy, which result in more defects that affect optical and electronic properties.

### 3.3. Sol–Gel Method

Scientists made early discoveries in mid-19th-century silica gel studies [58], which led to the sol–gel method, and this was further developed by the studies of Ostwald and Rayleigh [59] and adapted for perovskite synthesis today thanks to Belik and Yi [60]. Here, metal alkoxides are hydrolyzed and then linked through polycondensation, resulting in a colloidal solution (sol) that develops into a gel network [61]. Controlling pH, temperature, and precursor composition leads to different nanotube and NP morphologies, especially with sizes in the range of 10–100 nm [62]. The Pechini approach, with the assistance of citric acid and similar chelating agents, ensures that cations are evenly distributed and that perovskites can be crystallized at lower temperatures (from 600 to 1000 °C). This molecular approach offers excellent control over molecule ratios, is very pure, and requires less energy than solid-state reactions (<220 °C). Catalytic and optoelectronic properties are greatly improved by altering the structure of a catalyst or composite using this method [63]. However, difficulties in scaling up photosensitive materials and the time-consuming nature of the methods continue to limit their use [64].

Unlike hydrothermal or co-precipitation methods, the sol–gel technique produces uniform particle sizes and prevents significant clumping of NPs below 50 nm [65]. SrZrO_3_:Eu^3+^ hollow spheres gave rise to improved emission lifetimes and higher PLQY at elevated temperatures, an outcome of better crystallization [62]. Optimized gelation kinetics enhance the lifetime of the intrinsic charge carriers, thereby reducing non-radiative recombination, which is essential for light-emitting applications.

### 3.4. Hydrothermal Synthesis

Phase-pure perovskite materials were easily produced by hydrothermal synthesis in the 20th century because it operated at moderate temperatures of 150–240 °C in an aqueous solution [66]. The process starts by dissolving relevant chemicals in an alkaline solution, followed by an autoclave crystallization under controlled pressure and adjusted viscosity [67]. The desired size and faceting of NPs (200 nm to 2 μm) depend on their hydroxide concentration and medium viscosity, which decreases their tendency to cluster together [66]. The advantages of this method include the accurate measurement of components, high uniformity, and the ability to produce materials in large quantities (such as Na_0.5_Y_0.39_Yb_0.1_Er_0.01_TiO_3_) [67]. While solid-state techniques require high temperatures, hydrothermal synthesis does not, which reduces energy use and maintains the NPs shape [66]. Unfortunately, some issues still exist, especially with pH control and viscosity, processes that are often slow and do not easily remove BaCO_3_ from the mixture [67].

In making sub-200 nm particles with a narrow size distribution, hydrothermal synthesis is more effective than sol–gel and co-precipitation approaches [66]. By avoiding defects through control over crystallinity, PLQY is increased. For example, uniform CsPbBr_3_ PNCs have fewer pathways for excitation energy to be lost as heat, so their emission properties are enhanced [68]. Changes in interfacial strain and octahedral distortion made possible by this method also improve carrier recombination, enhancing the PLQY without direct intervention [69].

### 3.5. Solvothermal Synthesis

This method was initially introduced for perovskites in the mid-2010s. The solvothermal synthesis is useful for producing nanostructured hybrid and inorganic perovskites by controlling the reaction of salts with specific solvents at high temperatures (80–240 °C) [70]. To prepare the material, metal halides and organic precursors are dissolved in a mix of DMF and DMSO and then sealed in an autoclave to regulate crystallization rate and crystal growth [71]. CsPbBr_3_ nanoplatelets, with a thickness of 2.3 nm, were prepared using PbBr_2_ and solvothermal reactions, causing electrons to be confined and producing blue light with a wavelength in the spectral range of 459–467 nm [72]. Some important features are unmatched levels of crystallinity, the ability to scale up, and being compatible with FA_0.5_MA_0.5_PbI_3_-type mixed cation/halide systems [73]. Reducing the oxidative damage in their closed systems caused less surface damage on CsPbBr_3_ nanoplatelets, which increased their PLQY to nearly unity [72]. Yet, challenges remain in terms of solvent safety, extended reaction times (up to three days), and the presence of residual ligands that slow the transfer of charge in the film [73].

Unlike hot-injection or solid-state synthesis, solvothermal synthesis provides high control over producing NPs under 20 nm and single-crystal particles up to several micrometers, making 10–30 μm ferroelectric CsPbBr_3_ nanoplatelets a good demonstration [74]. The slow-paced crystallization leads to many defects being healed, resulting in higher PLQY. As a result, co-doped CsPbCl_3_ nanostructures show improved emission stability and tunable lifetimes [71]. However, the type of solvent can have a significant effect on the purity of separation process, with many aqueous systems relying on iodide inclusion to avoid precipitation of PbI_2_ [75].

### 3.6. Kinetically Controlled Space Confinement

The introduction of the KCSC method in 2020 allows for synthesizing phase-pure 2DPs with the desired layer thicknesses (*n* is 1–6, denoting the number of corner-sharing BX_6_ octahedral layers between organic spacer sheets) [76]. Using this method, the nucleation process is controlled by regulating the temperature so that the growth of the particles in small regions leads to a consistent transformation from Ruddlesden–Popper to Dion–Jacobson phases through intercalation [77]. KCSC outperforms hydrothermal and sol–gel techniques because it produces crystals with exact atomic composition, preventing random mixing of phases that are typical in these approaches [78]. Slow growth of the crystals for several minutes to hours allows the removal of any flaws and enhances PLQY due to fewer sites where non-radiative recombination occurs [77]. It has been observed that *n* = 3–5 systems of CsPbI_3_ exhibit a PLQY greater than 80% since dielectric confinement is weak and exciton binding is enhanced [79]. Even so, as temperature, precursor proportions, and various reaction conditions are closely controlled, it becomes hard to scale up the process [76].

According to a comparison (Table 2), KCSC performs better than conventional methods for producing sub-20 nm NPs or perovskites with more than five layers, since quantum features guide their optoelectronic behavior [80]. The literature has confirmed that controlling the growth space reduces the high interfacial stress, allowing for heterostructures with up to 11% mismatch [36]. Although the material quality is good, the use of DMF as a solvent is still toxic, and residual organic ligands remain, requiring an extra purification process [76].

### 3.7. Solid-State Route

The solid-state method for fabricating perovskites is a widely adopted approach involving the direct mixing and high-temperature treatment of solid precursor powders. This process starts with the thorough grinding of stoichiometric amounts of solid precursors such as metal oxides, carbonates, or nitrates, using a mortar or ball mill to achieve a uniform mixture. The blended powders are then calcined at elevated temperatures, generally between 800 and 1200 °C, for several hours in a furnace. This heat treatment facilitates diffusion and solid-state reactions among the components, resulting in the formation of the target perovskite crystal structure. Sonawane et al. [82] prepared Sr(Al_0.5_Nb_0.5_)O_3_ perovskite using conventional solid-state techniques, yielding materials with well-defined crystallinity and promising electronic properties, such as a Pb-free composition and a bandgap in the range of 1.3–1.6 eV. Such examples highlight the versatility of the solid-state method in producing functional perovskites for various applications.

These features enable efficient absorption across the Vis light spectrum, offering an environmentally responsible alternative to conventional Pb-based perovskites, such as CH_3_NH_3_PbI_3_. Furthermore, the strontium-based oxide offers outstanding thermal and chemical resilience, making it more stable under operating conditions. This intrinsic stability enhances its suitability for long-term stability in solar panels, where degradation is a persistent challenge. Overall, Sr-based perovskites provide a viable route toward safe, stable, and sustainable solar technologies [82]. The solid-state route is a straightforward and cost-effective route, well-suited for industrial-scale fabrication with well-defined crystal structures and minimum contamination. Besides the advantages, it requires prolonged high-temperature treatments (typically above 1000 °C), leading to high energy consumption, and a large particle size may be obtained.

### 3.8. Solution Process Method

This method is based on the preparation of a precursor solution by dissolving stoichiometric amounts of perovskite-forming compounds, such as metal halides (e.g., lead iodide, methylammonium iodide) or metal salts, into an appropriate solvent or solvent mixture [82]. Common solvents include DMF, DMSO, or gamma-butyrolactone (GBL), chosen for their ability to dissolve the precursors uniformly and influence film formation. Once a clear and homogeneous precursor solution is prepared, it can be deposited onto a substrate using coating techniques such as spin coating, dip coating, spray coating, or inkjet printing. Spin coating is particularly popular due to its simplicity and its control over film thickness. During the coating process, the solution spreads evenly across the substrate surface. After deposition, the wet film undergoes a drying step to remove the solvent, often assisted by mild heating or a controlled atmosphere. This drying step is critical in the solution process method, as it influences nucleation and crystal growth. Following drying, the film is subjected to an annealing process at moderate temperatures (typically between 100 and 150 °C) to promote the crystallization of the perovskite phase and improve film quality by enhancing grain size and reducing defects.

The solution process method offers several advantages, including low cost, scalability, compatibility with flexible substrates, and the integration of perovskites with conventional substrates, forming a new class of hybrid heterojunction for multiple applications. Indeed, it provides excellent control over the composition, morphology, and thickness of the perovskite films, which are essential parameters for optimizing device performance [82]. This method is extensively used in the fabrication of perovskite SCs, LEDs, PDs, and other optoelectronic devices.

Interfacial modifiers were metal-oxide NP buffer layers (PBLs), often ZnO, Al-doped ZnO (AZO), or SnO_2_, used to induce morphology smoothing, energy level alignment, and protection of the perovskite/transport stack during deposition of transparent-electrode modifiers. Glass substrates that were ITO-coated were solvent-cleaned and UV-ozone treated. An annealed hole-selective contact (e.g., PTAA) was spin-coated, annealed; a work-function modifier (e.g., PFN-Br) was solution-added where necessary to tune the ITO/HTL interface. The perovskite absorber was grown using either a one-step solution process or a two-step hybrid conversion, and annealed depending on the composition of choice, with an electron-selective coating (e.g., PCBM). PBL dispersion (ZnO/AZO/SnO_2_) was subsequently spin-coated (statically or dynamically) and annealed slightly to allow densification and binding without surpassing the thermal budget of perovskite. The ITO top contact was deposited by low-damage RF sputtering, with the PBL used as a sputter-shield/band-alignment layer. To establish the active area and minimize series resistance, metal grids or a specified aperture were added. To operate tandem ally, the semitransparent perovskite device was monolithically stacked on a silicon heterojunction (SHJ) bottom cell, with the PBL/ITO stack to provide a low-loss recombination contact, and to ensure optical transparency to harvest light at the rear. This methodology enabled the fabrication of fully solution-processed ST-PSCs, reaching a PCE of 18%, and tandem SCs, achieving efficiencies up to 25% [83]. This method provides a scalable and low-cost alternative to atomic layer deposition (ALD), which is slow and expensive. It improves the structural and electrical properties, yielding high PCEs in both ST-PSCs and tandem cells. Although the advantages of the solution process approach are significant, it also has some limitations, such as the fact that static coating may cause underlying damage, leading to pinholes and performance issues. Devices using SnO_2_ buffer layers underperformed due to weak UV protection and poor crystallinity. Rabhi et al. [84] synthesized the MAPbI_3_ perovskite via a one-step method, incorporating in situ PbI_2_ passivation. The MAPbI_3_ films show a tetragonal structure and a bandgap of 1.53 eV, enabling strong absorption. Stimulated devices based on these films achieve a PCE of 24%, which is further improved to 26% using optimized contact layers (AZO and IZO) instead of gold (Au). These findings highlight a simplified fabrication route for high-efficiency PSCs.

In summary, the solution process method combines the simplicity of solution chemistry with precise PTF deposition techniques to produce high-quality perovskite materials, making it a cornerstone of modern perovskite research and commercialization.

### 3.9. Microwave-Assisted Synthesis

Microwave-assisted synthesis is an experimental technique that has been shown to be a viable alternative to traditional hot-injection methods of PNCs since the microwave irradiation can provide rapid, volumetric (dielectric) heating, allowing rapid and reproducible temperature modulation and requiring typically only seconds to minutes to react under varied microwave power/time and precursor/ligand conditions; adjusting the nucleation/growth balance in such a system can be used to achieve batch-to-batch consistency and to access diverse morphologies [85]. As an example, Pan et al. [86] have described a rapid and efficient microwave approach to the synthesis of high-quality CsPbX_3_ (X = Cl^−^, Br^−^, I^−^) NCs, with shapes (nanocubes, nanoplatelets, nanorods), high PLQY of 75%, and histograms with narrow emission linewidths, and have shown their application in LED prototypes. The microwave protocols can also be purposefully adjusted to slow down growth in order to isolate intermediate growth phases to provide a mechanistic understanding of the CsPbBr_3_ NCs growth pathways.

### 3.10. Synthesis Methods and Device Applications

**Figure 7:**
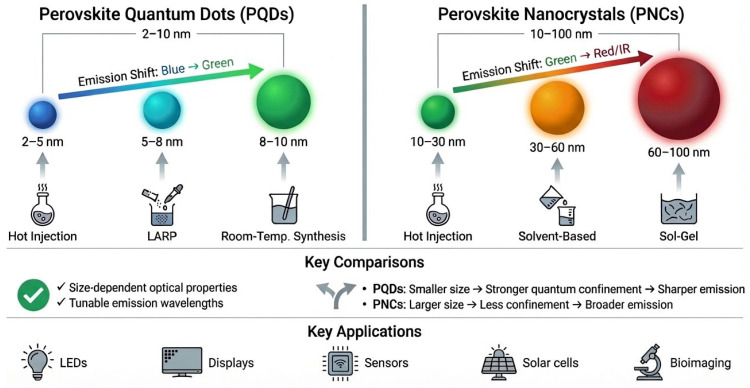
Size-dependent optical properties of PQDs and PNCs, highlighting emission shifts with varying particle sizes and the effects of different synthesis methods.

The optical properties of perovskite materials are highly dependent on their particle size, which can be precisely controlled with different synthesis techniques. Size control is essential for tuning the emission wavelength and optimizing the material performance for specific applications, such as LEDs, SCs, and displays. For instance, PQDs with size ranging 2–10 nm exhibit the quantum confinement effects that cause tunable emission, shifting from blue to green as the size increases, as shown in Figure 7. In contrast, PNCs of size ranging from 10 to 100 nm show broader emission spectra, with a shift from green to red/infrared, reflecting the reduced confinement in larger particles. Synthesis techniques, such as the hot-injection method, RT synthesis, and the sol–gel method, provide precise control over particle size, influencing both absorption and emission properties and ultimately enhancing the performance of perovskite-based devices.

## 4. Application of Perovskite Crystals, Quantum Dots, and Two-Dimensional Structures

The perovskite crystals, PTFs, PNCs, PQDs, and 2DPs vary greatly in their dimensionality, morphology, and quantum confinement; however, they have a common group of optoelectronic properties that binds their relevance to similar application spheres. These are tunable direct band gaps that are high in absorption coefficient, high light–matter interaction, defect-tolerant electronic structures and high PLQYs. The applications in the current section, namely, LEDs, SCs, PDs, lasers, and biomedical luminescence imaging, are the most technologically developed and explored environments in which these perovskite-based materials have exhibited exceptional and comparable performance. Although not all forms are as optimized to all applications, complementary functionality is made by the property to be color pure in the case of PNCs and PQDs, and in quantum confinement photonics, as well as the ability of 2DPs to provide improved environmental stability and excitonic control. This part thus highlights typical uses, in which the structure–property–performance relationships of various perovskite structures and applications can best be demonstrated. Finally, the possible application of perovskite nanomaterials is explicitly established.

### 4.1. Light-Emitting Diodes

**Figure 8:**
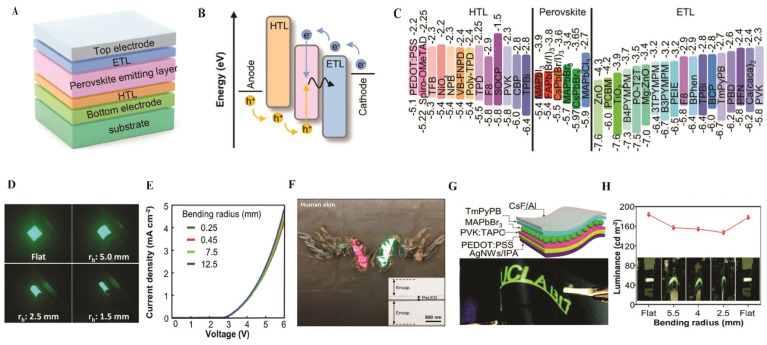
(**A**) Typical device structure. (**B**) Light emission mechanism of PeLEDs. (**C**) Energy level alignment of various materials used as HTLs, perovskites, and ETLs in the PeLEDs [87]. Copyright 2014, *Springer Nature*. (**D**) Digital photograph of PeLEDs at different bending radii. Reproduced with permission [88]. Copyright 2019, *American Chemical Society*. (**E**) I-V characteristic of PeLEDs at different bending radii. (**F**) Digital photograph of multicolor transfer-printed PeLEDs on human skin; inset displays the FIB image. Reproduced with permission [89]. Copyright 2022, the *American Association for the Advancement of Science*. (**G**) Device structure (top) and optical image (bottom) of the flexible MAPbBr_3_ NCs PeLED composed of AgNW-polymer composite electrode. (**H**) Luminance of the flexible PeLED at various bending radii. Reproduced with permission [90]. Copyright 2017, the *Royal Society of Chemistry*.

PeLEDs are devices used for the conversion of electrical energy to light. In parallel, perovskite crystals and PTFs have been widely adopted as emissive layers in light-emitting applications. Hybrid organic–inorganic PTFs, such as MAPbBr_3_ and FAPbI_3_, exhibit a high absorption coefficient (>10^5^ cm^−1^), a direct bandgap tunable between 1.5 and 3.0 eV, and a high PLQY above 90% [26,28]. Their compatibility with low-temperature processing offers a pathway toward low-cost and scalable fabrication. TF PeLEDs using mixed-cation composition like FA-Cs blends have achieved EQE of 22% [30]. Advances such as the introduction of 2DP layers, surface passivation with insulating polymers, and compositional engineering greatly enhanced both efficiency and device stability (Figure 8).

The basic device architecture and operating principles are summarized in Figure 8. A type of PeLED consists of a substrate, transparent electrodes (anode and cathode), carrier transport layers (electron transport layer (ETL), and hole transport layer (HTL), as well as the perovskite emissive layer, as shown in Figure 8A. The perovskite is sandwiched between the ETL and HTL, forming a multilayer heterojunction that enhances the charge injection and confinement, thereby boosting the light emission. Depending on the stacking order, PeLEDs can be fabricated in conventional bottom-emitting (HTL-perovskite-ETL) or inverted top-emitting (ETL-perovskite-HTL) configurations [87]. Figure 8B depicts the light-emitting mechanism. Figure 6B illustrates the light-emitting mechanism: electrons injected from the cathode through the ETL occupy the conduction band of the perovskite, while holes injected from the anode through the HTL occupy the valence band. The recombination of these charge carriers in the perovskite layer results in photon emission. Radiative recombination of these confined carriers generates photons whose wavelength is determined by the perovskite bandgap. Figure 8C summarizes the energy levels of commonly used transport and emissive materials, emphasizing that unbalanced charge injections, typically due to higher electron mobility in ETLs compared to hole mobility in HTLs, can limit device performance. Therefore, it is essential for effective transport layers to have (i) proper energy-level alignment with the perovskite to minimize injection barriers, (ii) balanced carrier mobilities, and (iii) processing compatibility to avoid damaging the perovskite layer [87]. Mechanical flexibility is a significant prerequisite for emerging PeLED technologies. Figure 8D shows the side-view images of emissive flexible PeLEDs under different bending radii, demonstrating that cautious structural design allows the devices to sustain mechanical stress while maintaining stable light output [88]. Ultrathin and skin-attachable PeLEDs further push the boundaries of wearable displays. As shown in Figure 8E,F, transfer-printed PNC layers enable devices with turn-on voltages below 3.0 V and EQEs of up to ~6.2%. These ultrathin (~300 nm) devices exhibit reliable electroluminescence even when bent at radii ranging from 250 nm to 12.5 mm. Photographs of multicolor PeLEDs attached to human skin highlight their potential for conformal, lightweight, and wearable optoelectronics. Cross-sectional transmission electron microscopy (TEM) images (inset of Figure 8F) reveal the total thickness of ~2.6 μm, confirming the feasibility of transfer-printed, skin-compatible designs [89].

Flexible substrates and electrodes are also critical for device durability. Figure 8G illustrates a sandwich-structured flexible CH_3_NH_3_PbBr_3_ QD LED fabricated on PET substrates, which are brittle and prone to failure under mechanical stress. AgNW polymer composites provide enhanced flexibility and smooth conductive surfaces that mitigate electrical shorting. This enables mechanically robust, high-performance flexible LEDs [90]. Figure 8H quantifies the mechanical resilience of CH_3_NH_3_PbBr_3_ QD-based LEDs by monitoring luminance under different bending radii. When driven at 3.5 V, the luminance decreased moderately (from 184 cd/m^2^) as the bending radius decreased to 2.5 mm, corresponding to ~20% loss. Significantly, the devices recovered ~87% of their initial luminance upon release, confirming their durability and reversibility under repeated mechanical deformation [90].

The first PNC-LEDs were reported by Schmidt et al. [52] in 2014, using MAPbBr_3_ PNCs as emitters. However, the luminescence of the device was extremely low (less than 1 cd/m^2^), and the work opened a new window of opportunities for PNCs electroluminescent LEDs. In 2015, Song et al. [91] first fabricated all-inorganic CsPbX_3_ PNC-based LEDs, with an EQE in the range of 0.1–0.4% for orange, green, and blue emissions. PNCs, such as CsPbBr_3_ and CsPbI_3_, emerged as promising emissive materials. Their unique properties, including narrow emission linewidth (~20 nm), tunable bandgap (1.5–3.0 eV), and remarkably high PLQYs greater than 90%, make them ideal for high-performance PeLEDs. Current devices now achieve an EQE exceeding 20% (Table 3) for green emissions [34]. For example, CsPbBr_3_ NC-LEDs have reached EQE of 21% with enhanced operational stability exceeding 100 h. The soft ionic lattice of perovskites also facilitates defect self-healing, enhancing both brightness and device lifespan [92].

PQDs further enhanced the advantages offered by nanocrystalline materials, combining the advantages of quantum confinement and PTF compatibility. CsPbBr_3_, CsPbI_3_, and CsXY halide-based PQDs have strongly tunable emission, with deep blue to NIR (410–700 nm) spectra, with narrow spectral widths and PLQYs up to 90% [41]. Their densities are very low, and they exhibit rapid radiative recombination rates (less than 20 ns) and efficient charge transport properties, which make them highly effective in LED applications. Recent PQLEDs have an EQE of more than 23% for green and 14% for the red light emission, with radiance exceeding 750 W/(sr·m^2^) [92]. Notably, PQDs exhibit low nonradiative losses and lower Auger recombination rates compared to conventional QD systems. Therefore, they can be used to produce ultra-bright and low-threshold sources of light.

Two-dimensional hybrid perovskites such as (PEA)_2_PbI_4_ have also garnered significant interest for light-emitting applications [97]. 2DPs benefit from naturally occurring quantum-well structures that enhance carrier confinement and radiative recombination. Even 2DP-LEDs have achieved an EQE above 14% with high stability, attributed to their layered structure that suppresses ion migration and enhances environmental durability. The anisotropic optical properties and flexible integration potential of 2D materials open exciting avenues for the development of transparent, wearable, and ultrathin optoelectronic devices [6,19,96].

### 4.2. Solar Cells

Perovskite materials have revolutionized photovoltaic technology due to their outstanding optoelectronic properties, including high absorption coefficients (>10^5^ cm^−1^ for MAPbI_3_), tunable bandgaps (1.2–3.0 eV), and long charge carrier diffusion mobility exceeding 1 μm [25]. These characteristics arise from the intrinsic flexibility of the ABX_3_ crystal structure [98]. This structural tenability enables a vast compositional space, allowing researchers to tailor properties for a range of optoelectronics applications. In photovoltaics, PSCs have witnessed a meteoric rise in performance, with PCE = 3.8% reported in 2009 by Kojima et al. [99] for the first time, while Shi et al. [100] reported a PCE = 12.7% for a non-sensitization type SC made of CH_3_NH_3_PbI_3−x_Cl_x_.

The most developed and widely adopted configuration is PTF, which serves as the active absorbing layer in PSCs. Compositional engineering has significantly advanced PTF, resulting in dramatic improvements in both efficiency and long-term thermal stability. For instance, the triple-cation formulation Cs_0.05_(FA_0.83_MA_0.17_)_0.95_Pb(I_0.83_Br_0.17_)_3_ developed by Saliba et al. [30] stabilized power output exceeding 21.1%. To bridge the gap between lab-scale achievements and industrial scalability, a new deposition method, blade coating, has emerged. Blade-coated devices have recently reached an efficiency of 22.3% [3]. Chen et al. [101] reported a PCE of 25.5% for blade-coated halide perovskite films.

**Figure 9:**
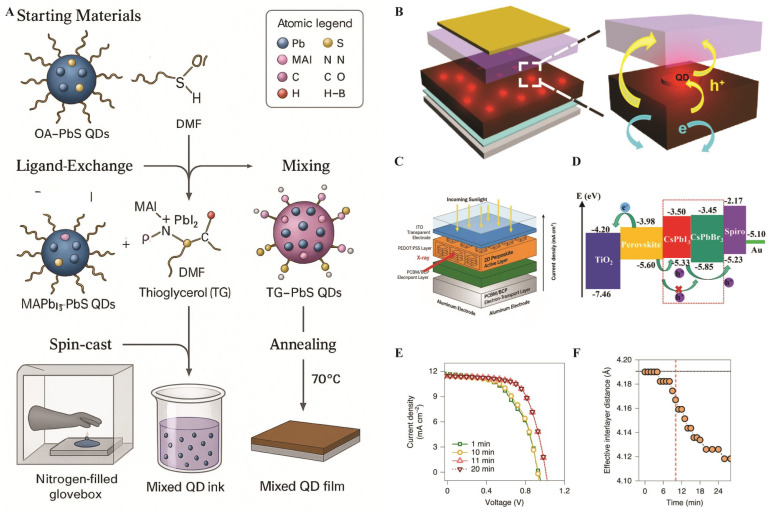
(**A**) Schematic illustration of the synthesis of the PSC mixed QD film. (**B**) PSC device structure [102]. Copyright 2016, *American Chemical Society*. (**C**) Device architecture used for fabricating SCs [105]. Copyright 2022, *Nature Nanotechnology*. (**D**) Energy diagrams of each material in PSC device [103]. Copyright 2018, *NPG Asia Material*. (**E**) Evolution of the current–voltage characteristics of the SC under constant AM 1.5 one-sun intensity illumination, measured in the DJ device during in situ GIWAXS diffraction. (**F**) Corresponding structural changes. The vertical dashed line indicates the time at which SC performances increase drastically [105].

To further improve the performance of the device, researchers have explored the incorporation of QDs into PTFs. As illustrated in Figure 9A, introducing MAPbBr_3−x_I_x_ QDs into the perovskite absorber establishes a stepwise hole-transfer pathway that surpasses recombination and enhances current density and PCE. The corresponding device layout, shown in Figure 9B, consists of transparent conducting oxide, a compact electron-transport layer, the perovskite absorber with an interfacial or mixed-QD layer, hole-transport materials, and a metal electrode. This QD layer not only provides energy-level alignment but also improves device stability, particularly when inorganic CsPbI_3_ QDs are used [102]. The energy-level diagram in Figure 9D, however, shows how QDs form cascade alignment between the perovskite valence band and the hole transport material, accelerating charge extraction and reducing recombination losses. Such improvements lead directly to higher open-circuit voltage and enhanced device efficiency [103].

After these developments, intrinsic stability issues have been a driving force in investigating 2DPs. These layered materials, made of organic and inorganic sheets, which are alternated, offer increased environmental resistance with no loss in functional optoelectronic characteristics. Shen et al. [104] reported that (BA)_2_(MA)_4_Pb_5_I_16_ provided a good quantum confinement, increased thermal stability, 22% PCE, and great moisture resistance.

Current attempts have been made on in-operando characterization, which allows direct correlation between structural evolution and device performance. Figure 9C illustrates integrated grazing-incidence wide-angle X-ray scattering (GIWAXS) and simultaneous current-voltage (I-V) monitoring under continuous illumination. These measurements, as shown in Figure 9E, summarize the results of analyses of Dion–Jacobson and alternating-cation perovskites. The results indicate that both materials exhibit abrupt performance improvements after several minutes of sustained illumination, whereas Ruddlesden–Popper phases show insignificant changes. This could be attributed to structural contraction, as shown in Figure 9F, wherein illumination results in out-of-plane lattice shrinkage, causes vertical charge transport, and leads to a tremendous improvement in carrier mobility [105].

Parallel to these developments, PNCs have emerged as highly tunable and solution-processable alternatives. Due to quantum confinement effects, PNCs offer bandgap tunability through their size and composition variation. The landmark synthesis of CsPbBr_3_ PNCs by Protesescu et al. [14] demonstrated a PQLY exceeding 90%, setting the stage for optoelectronic applications beyond photovoltaic. In PSCs, a recent study has shown that the CsPbCl_3_ PNCs exhibit enhanced structural stability, resistance to degradation efficiency, lower trap density (1.5 × 10^−16^ to 9.8 × 10^−15^ cm^−3^), and increased activation energy (0.47 to 0.75 eV). Therefore, the PCE is increased from 22 to 25%, and there is an ~8-times improved T_80_ lifetime operation under UV irradiation and an ~7-times improved T_80_ lifetime of the heat–light test [94].

PQDs, which are ultrasmall PNCs (less than 10 nm) that exhibit a strong quantum confinement effect, stand at the leading position in advanced optoelectronics. Their narrow emission spectra and size-tunable absorption make them an ideal candidate for SC applications. Swarnkar et al. [13] first demonstrated efficient PSCs based on CsPbI_3_ PQDs, with subsequent improvements involving bidentate ligand passivation to suppress trap-assisted recombination, pushing efficiencies to 16.6%. Moreover, bandgap tunability via halide mixing, such as CsPb(I_1−x_Br_x_)_3_, has enabled high PCE and strong quantum yield, making it suitable for SC applications [106]. Recently, Jeong et al. [95] improved the structure stability and increased the PCE to 23% by doping Mn^+2^ into CsPbCl_3_, as shown in Table 3. In addition, the use of hydrophobic ligands improves cell stability, maintaining 88% of the initial PCE after 500 h of storage in ambient air by inhibiting moisture penetration.

Several challenges remain to be addressed to realize the commercial potential of perovskite solar technologies fully. Among these challenges, Pb toxicity is the main obstacle, which has spurred interest in Pb-free alternatives.

### 4.3. Photodetectors

PD is a device that converts the optical signal into an electrical signal. As a new generation of optoelectronic materials, organic–inorganic hybrid and all-inorganic perovskite materials have great potential in the development of PDs because of their high absorption efficiency and high carrier mobility. Therefore, a very thin layer of perovskite film can strongly absorb light. This allows photogenerated carriers to travel a very short distance, resulting in a rapid photoresponse. The key parameters that characterize the quality of PD are photocurrent (*I_pc_*), responsivity (*R* = *I_pc_*/*P*), specific detectivity (D*), noise equivalent power (NEP), linear dynamic range (LDR), and response speed. The design of the device structure and the selection of the interface material will influence the performance of PDs [107]. The Guan research group has designed self-powered flexible PDs through vacuum filtration integration of MAPbBr_3_ QDs within sulfated cellulose nanofiber (S-CNF) papers. The Au/MQDs@10/CNF/Pt device achieved rapid response times of 290/510 ms, along with D* = 1.58 × 10^8^ Jones and *R* = 0.19 mA/W. The device remained operable at >91% of its initial responsivity when stored under 60% humidity conditions for 60 days and withstood 500 bending cycles without any structural or performance degradation. Excessive MQD incorporation deteriorated the device performance, yet the S-CNF framework maintained stability through Pb^2+^-sulfate bonding. This paper-based detection device could be safely incinerated within 0.5 s for environmental benefits. Perovskite optoelectronics achieve high stability through a technological approach that also provides mechanical flexibility [108].

**Figure 10:**
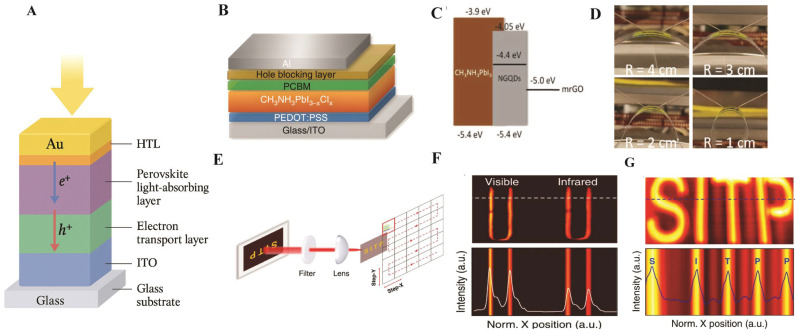
(**A**) Schematic illustration of perovskite PDs. (**B**) Device structure of the hybrid PD. (**C**) Energy diagram of the PD under a slight reverse bias [109]. Copyright 2014, *Nature Communications*. (**D**) UV-Vis absorption spectra of the PD without the hole-blocking layer and the Al electrode [108]. Copyright 2024, *Royal Society of Chemistry*. (**E**) Schematic of the image scanning system and actual imaging for the OIHP PD. (**F**) NIR imaging results of the heated coil. (**G**) Imaging of letter graphics under LED illumination [110]. Copyright 2020, *Light: Science and Applications*.

Figure 10A highlights several representative structural and functional demonstrations of perovskite PDs. Figure 10B,C show a hybrid CH_3_NH_3_PbI_3−x_Cl_x_ device with PEDOT:PSS and PCBM transport layers, while interfacial buffers such as BCP and PFN effectively block holes and suppress dark current (*I_dc_*), thereby enhancing D* [109]. Figure 10D illustrates the flexibility of MQD/cellulose nanofiber-based PDs, which maintained stable performance during bending tests, emphasizing their suitability for wearable electronics [108]. Figure 10E–G demonstrate broadband imaging, including Vis-NIR imaging of a heated coil and successful reconstruction of letter graphics under LED illumination [110]. These results showcase the dual-band recognition ability and practical imaging potential of perovskite PDs.

Jeong et al. [111] introduced a novel class of 0D-2D hybrid PDs, combining PQDs such as CsPbI_3_, Cs_x_FA_1−x_PbI_3_, and FAPbI_3_ with bilayer MoS_2_, offering tunable bandgap. The coupling between PL and the built-in potential, which exceeds 1.0 eV, led to a high *R* and D* exceeding 10^13^ Jones. The PD’s selectivity stems from its spectral response range, which matches the QD absorption spectrum (600–800 nm). Under optimized operation, Cs_2_FA_1−x_PbI_3_/MoS_3_ PDs show sublinear power-law behavior: *I_pc_* scales with incident optical power as *I_pc_* ≈ *P*^α^, with α = 0.39–0.46. Consequently, *R* ≈ *P*^α−1^ ≈ *P*^−0.53^ − *P*^−0.60^. Thanks to the uniformity of MoS_2_ prepared by MOCVD, the device supports the simultaneous advantages of PQD absorption and 2D carrier transport.

Wang et al. [112] synthesized Pb-free CsSnX_3_ PQDs by varying the precursor concentration of Cl, Cl/Br, and Br to produce particles with adjustable emission wavelengths ranging from 425 to 525 nm. The PLQYs increased from 5.1 to 16.2%, while defect density decreased from 4.25 × 10^16^ to 1.87 × 10^16^ cm^−3^ after substituting Cl^−^ with Br^−^. The PD made of CsSnBr_3_ reached *I_pc_* of 122 nA at 3 V and D* of 1.27 × 10^11^ Jones due to low defect density. Such a device achieved ultralow *I_dc_* = 10^−2^ nA with response time less than tens of milliseconds and displayed superior performance than ZnO devices. I-V measurements as a function of temperature displayed strongly insulating characteristics under dark (exceedingly small leakage current) conditions. The bandgap, and hence the absorption edge, could be tuned by replacing various halide anions in the lattice (e.g., Cl^−^, Br^−^, I^−^), allowing the device to generate a measurable photoresponse between 250 and 800 nm and providing an UV-to-Vis response with low *I_dc_*.

### 4.4. Lasers

The emergence of novel optoelectronic materials with exceptional light-emitting and gain properties has significantly spurred advancements in semiconductor lasers. Among the most promising materials for next-generation laser technologies are MHPs, 2D layered structures, PQDs, and PTFs. These materials offer distinct advantages, such as tunable emission spectra, high PLQY, low lasing thresholds, and compatibility with solution-processable fabrication techniques. Hybrid organic–inorganic PNCs, typically with ABX_3_ structure, have shown exceptional optical characteristics. The inherent high PLQY, sharp emission linewidth, high absorption coefficient, and tunable bandgap make perovskites, particularly methylammonium lead halides (e.g., CH_3_NH_3_PbBr_3_), highly favorable for lasing applications. These materials have low Auger recombination and high optical gain; these are necessary to attain effective population inversion and stimulated emission [113].

Closely, PTFs have also come up as a feasible and scalable laser integration architecture. Unlike single crystals, PTFs can be produced on a broader scale by photolithography and in more flexible device configurations. They can be deposited through a variety of methods, including spin coating, vapor deposition, and inkjet printing. PTFs, such as those made from MAPbBr_3_ or CsPbBr_3_, have demonstrated amplified spontaneous emissions (ASE) and lasing under pulsed optical excitation, with thresholds as low as a few μJ/cm^2^ [114]. Soriano-Díaz et al. [115] produced Ni(ACO)_2_ TFs containing MAPbBr_3_ PNCs with enhanced spontaneous emission and lasing. This property of the materials, threshold energy, makes them a good candidate for use in laser applications, especially in optical waveguiding systems. The morphology, grain boundary, and optical cavity design of the film are essential in determining the lasing performance. The latest development in TF processing has resulted in the production of smooth and low-defect film with enhanced thermal and photostability, thereby mitigating one of the major drawbacks of the first perovskite lasers. Furthermore, the feasibility has been demonstrated with vertical cavity surface emitting lasers (VCSELs) and distributed feedback lasers, which rely on PTFs, integrating these materials into miniaturizable and tunable photonic platforms [116].

Another highly useful medium for lasers is PQDs, such as CsPbBr_3_. Their size-dependent tunable emission, high oscillator strengths, and enhanced radiative recombination rates are the results of quantum confinement effects in these PNCs. PQDs exhibit low-threshold lasing in various microcavity designs due to their high PQLY, which exceeds 90%, as well as the narrow linewidth of their emissions. Lasing-stable CsPbBr_3_ QDs have been pumped by optical means in resonant cavities, with a threshold of 5 μJ/cm^2^ [93]. Moreover, they can be engineered to have their surface chemistry and control doping, offering additional possibilities for optimizing lasing performance and lifetime.

Two-dimensional materials, specifically monolayer transition metal dichalcogenides and layered 2DPs, have potential for miniaturized laser devices. These materials exhibit large quantum confinement, resulting in intense excitonic behavior with binding energies of hundreds of meV, which facilitates efficient radiative recombination at RT [117]. Furthermore, 2DPs such as (C_4_H_9_NH_3_)_2_PbBr_3_ exhibit natural quantum-well structures, which enhance exciton confinement and provide high photostability. These layered structures offer solutions for processable fabrication, environmental resilience, and tunable emission, making them suitable for both vertical and planar laser geometries [118].

### 4.5. Biomedical Luminescence Imaging

Biomedical luminescence imaging, which encompasses fluorescence, bioluminescence, and PL, plays a crucial role in noninvasively visualizing cellular processes, disease pathophysiology, and drug responses in clinical and research applications [119,120,121]. CsPbBr_3_ and CsPbI_3_ are examples of lead-halide PNCs, which are grown by hot-injection or ligand-aided reprecipitation to yield 5–15 nm particles. Colloidal stability in aqueous media is improved by surface passivation with ligands such as OLA or by encapsulation in silica or polymers, e.g., poly(maleic anhydride-alt-1-octadecene), producing zeta potentials of about −40 mV. PNCs have size-tunable bandgap ranging 1.71–3.2 eV, PLQYs of 80–95%, PL lifetimes (10–100 ns), and low trap densities (<10^16^ cm^−3^) [122]. As an example, the polymer-encapsulated CsPbBr_3_ PNCs have a PLQY of ~84% at 518 nm (FWHM of ~18 nm). Mohapatra et al. [123] reported the CsPbBr_3_@SiO_2_ PNCs with an improved PLQY of up to 85%. These PNCs act as an efficient fluorescent probe, enabling them to serve as a biocompatible sensor for the detection of Hg^2+^ ions inside the mammalian 3T3L-1 cells through fluorescence imaging.

Sun, Y. et al. [124] studied the neurotoxicity caused by the CsPbBr_3._ In vivo, PNCs of CsPbBr_3_ (~40–50 nm) were intranasally administered into mice (10 L, 25 mg/kg, every other day, 28 days). Pb was built up through olfactory ways, particularly the hippocampus, leading to cognitive/behavioral deficits, neuronal apoptosis, gliosis, oxidative stress, and Ca^2+^ dysregulation. Biotransformation produced Pb salts; the toxicity was more than that of Pb acetate. Pb-free solutions or fail-safe encapsulation with fast clearance are required due to translation demands.

**Figure 11:**
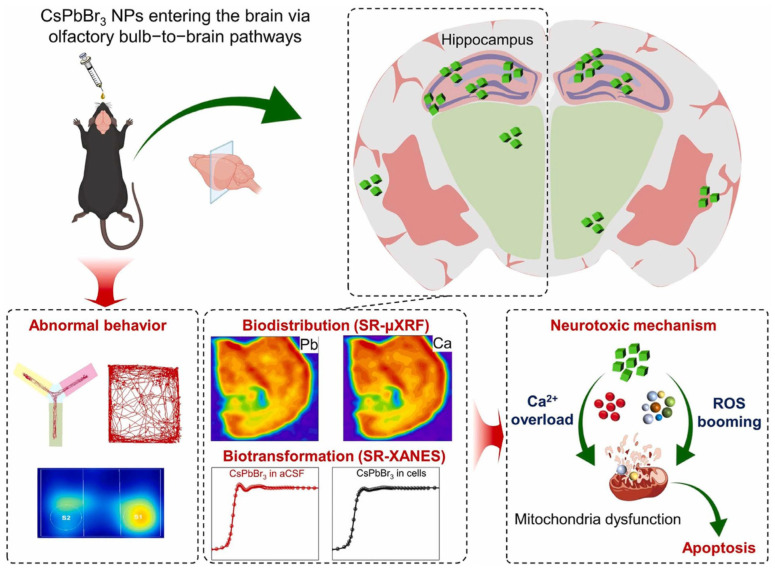
A schematic diagram of the translocation, biotransformation, and biodistribution-related neurotoxicity of CsPbBr_3_ PNPs [125]. Copyright 2023, *Nano Today*.

As demonstrated in Figure 11, mice intranasal exposure to CsPbBr_3_ PNPs displays an obviously hippocampus-dependent behavioral disorder. To understand the biodistribution and biotransformation of CsPbBr_3_ PNPs, advanced synchrotron radiation (SR)-based XANES and SR-μXRF techniques are powerful to illustrate the dynamic processes of CsPbBr_3_ PNPs neurotoxicity in time and space. Due to the advantages of high spatial resolution, high sensitivity, high accuracy, low matrix effects, and non-destructiveness, SR-based analytical techniques have become extremely valuable tools for exploring the interactions of the NP/biological interface. Here, SR-μXRF can in situ study the Pb accumulation in the brain, while SR-XANES can reveal the biotransformation of Pb in artificial cerebrospinal fluid (aCSF). CsPbBr_3_ PNPs induced cell apoptosis through upregulating reactive oxygen species and inducing Ca^2+^ overload [125].

Homogeneous emission, as well as waveguide capability, can be achieved with PTFs, prepared via spin-coating or vapor deposition, and microstructures, prepared by nanoimprinting or laser patterning, because of their high refractive indices (1.925–2.5). Through greater reduction in waveguide losses, patterned films have greater external PLQY encompassing 25–60%. CsPbBr_3_ films on microfluidic platforms allow imaging with guided-light PL with subcellular resolution in tissue models. Their application within implantable devices is still investigational in vivo, although there are prospects of tissue monitoring in real time.

**Figure 12:**
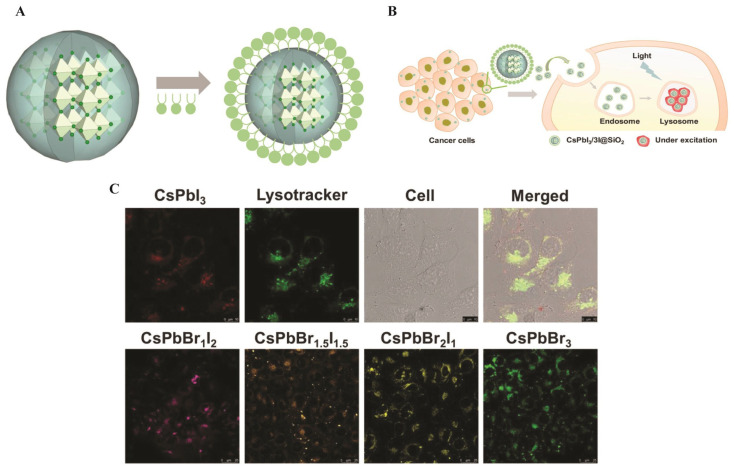
(**A**) Illustration of phospholipid encapsulation to prepare CsPbI_3_/_3_I@SiO_2_ phospholipid micelles. (**B**) Scheme of the phospholipid micelles for bioimaging. (**C**) CLSM images of MDA-MB-231 cells upon incubation with the phospholipid micelles; all the scale bars were 10 µm. CLSM images of MDA-MB-231 cells upon incubation with CsPbBr_3_, CsPbBr_2_I_1_, CsPbBr_1.5_I_1.5_, and CsPbBr_1_I_2_ QDs; all the scale bars were 25 µm [126]. Copyright 2022, *Wiley Online Library*.

PQDs, such as CsPbBr_3_ and CsPbI_3_ (<10 nm), leverage quantum confinement for emission tunability (450–1700 nm) and high oscillator strengths (10^−1^–10^0^). Surface coatings such as PEG or phospholipids, ensure biocompatibility (representative in Figure 12), with a zeta potential of −30 to −50 mV. CsPbBr_3_/NaYF_4_:Yb, Tm PQDs enable NIR upconversion under 980 nm excitation, with a PLQY of 0.25% for the upconverted emission (292–478 nm) and 23% under 365 nm excitation, retaining 80% intensity of the initial intensity after 6 h in ethanol.

Song et al. [126] reported that the perovskite QDs encapsulated in phospholipid-micelles and dually passivated delivered bright multicolor fluorescence (510–680 nm) with superior aqueous stability and a high PLQY of ~93%, while exhibiting consistently low cytotoxicity across the concentrations tested. They targeted lysosomes via confocal bioimaging in MDA-MB-231 cells, as illustrated in Figure 12. Figure 12A demonstrates that phospholipid encapsulation forms a stable micellar shell around perovskite cores, facilitating efficient endocytosis and lysosomal localization in MDA-MB-231 breast cancer cells (Figure 12B) [126]. Confocal laser scanning microscopy (CLSM) images reveal distinct intracellular fluorescence corresponding to PQD accumulation in lysosomes, as verified by colocalization with LysoTracker dye, as shown in Figure 12C. Moreover, a compositional series of mixed-halide PQDs (CsPbBr_3_, CsPbBr_2_I_1_, CsPbBr_1.5_I_1.5_, and CsPbBr_1_I_2_) produced well-resolved emission colors from green to red, confirming precise halide-dependent spectral tunability for multicolor imaging [126].

**Figure 13:**
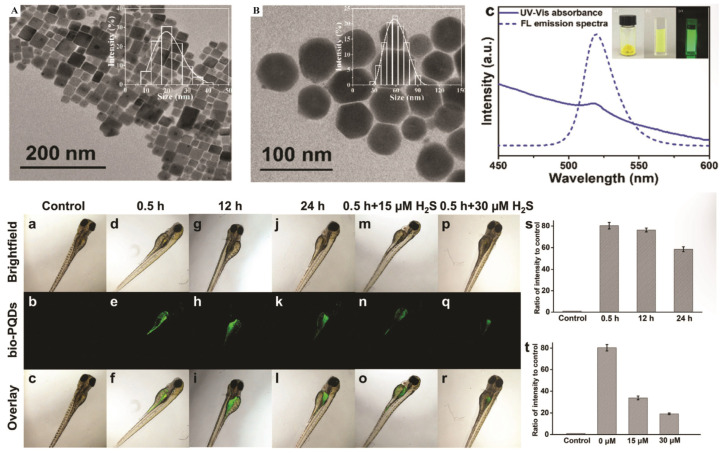
Characterization of bio-PQDs. TEM image of (**A**) PQDs in toluene (inset: size distribution of the PQDs determined by DLS) and (**B**) bio-PQDs in water (inset: size distribution of the bio-PQDs determined by DLS); (**C**) UV-Vis absorption (solid line) and PL emission spectra (dashed line) of the bio-PQD solution (inset: photographs of the bio-PQD powder, bio-PQDs in water under room light and UV light). In vivo H2S-responsive PL imaging of bio-PQDs. Confocal microscopy images of zebrafish after incubation (**a**–**c**) without or with bio-PQDs (100 µg/mL) for 0.5 h (**d**–**f**), 12 h (**g**–**i**), and 24 h (**j**–**l**) and treated with bio-PQDs for 0.5 h followed by 15 μM (**m**–**o**) or 30 μM H2S (**p**–**r**); quantification of the PL intensity per unit area for the zebrafish (**s**) treated with bio-PQDs for different time periods (**t**) and treated with or without H_2_S. Error bars represent the standard error derived from five repeated measurements [127]. Copyright 2021, *Royal Society of Chemistry*.

In vivo, NIR-II PQDs achieve ~1 cm imaging depths in zebrafish for H_2_S monitoring, with detection limits below 10^5^ cells/mL [127]. The hydrophobic polymer encapsulants not only suppress water-induced degradation but also deactivate surface trap states and can even improve the PLQY in certain situations. Along with these developments, PQDs have already found limited application in noninvasive biosensing/imaging, e.g., polymer-caged PQDs have been used to allow real-time tracking of the levels of hydrogen sulfide (H_2_S) in live cells and throughout zebrafish over prolonged (Figure 13) [127].

As depicted in Figure 13, TEM confirms the uniform cubic morphology of PQDs dispersed in toluene (Figure 13A) and their well-dispersed polymer-caged counterparts (bio-PQDs) in water (Figure 13B), both exhibiting narrow size distributions. The UV–Vis absorption and PL emission spectra in Figure 13C reveal a strong emission centered near 520 nm, with stable intensity under ambient and UV illumination, demonstrating the optical robustness of encapsulated PNCs [127]. In vivo fluorescence imaging in zebrafish embryos (Figure 13a–r) shows distinct spatiotemporal uptake of bio-PQDs after 0.5, 12, and 24 h of incubation, with green luminescence clearly localized along the intestinal and vascular regions. Subsequent exposure to 15 and 30 µM H_2_S (Figure 13m–r) induces a concentration-dependent enhancement in fluorescence, confirming the selective H_2_S-responsive behavior of the nanoprobes. Quantitative analyses (Figure 13s,t) further validate time- and dose-dependent increases in PL intensity, with high reproducibility across five replicates.

**Figure 14:**
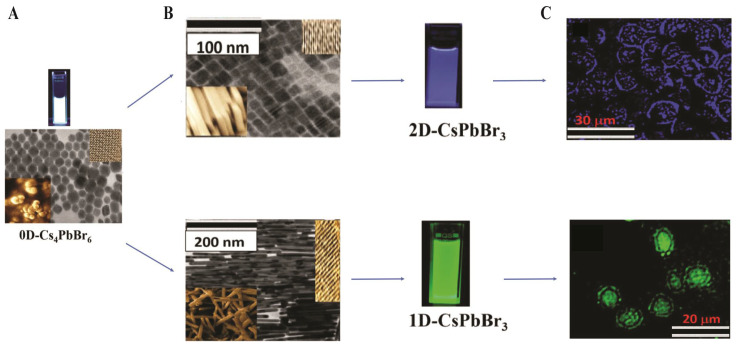
(**A**,**B**) TEM image, inserted HRTEM image, inserted SEM image, and photograph of water-resistant 1D, 2D, and 3D CsPbBr_3_ PNCs derived through an interfacial conversion from 0D Cs_4_PbBr_6_ PNCs using different amounts of water. (**C**) Two-photon luminescence image from PR(−) ER(−) HER-2(−) MDA-MB-231 breast cancer cells, which are attached to anti-AXL antibody-conjugated 2D CsPbBr_3_ nanoplatelets. Two-photon luminescence image from HER-2(+) SK-BR-3 breast cancer cells, which are attached to anti-HER-2 antibody-conjugated 1D CsPbBr_3_ nanowires [128]. Copyright 2020, *American Chemical Society*.

2DPs, (RNH_3_)_2_PbX_4_, feature quantum-well structures with >200 meV exciton binding energies, offering enhanced stability. They exhibit PLQYs of up to 45% and FWHM of 10–15 nm. In vitro, (PEA)_2_PbI_4_ films enable cell–substrate imaging at ~520 nm, retaining >80% PL intensity after 24 h in serum. Antibody-functionalized 2D CsPbBr_3_ nanoplatelets achieve selective two-photon imaging of breast cancer cells in the NIR window, with >86% PL intensity after 35 days in water; representative morphologies (1D/2D/3D) and two-photon cell images are provided in Figure 14 [128].

The morphology and dimensional control of CsPbBr_3_ PNCs were achieved through interfacial conversion of 0D Cs_4_PbBr_6_ precursors by varying water content, producing 1D nanowires, 2D nanoplatelets, and 3D nanocubes with distinct emission colors (Figure 14A,B). The 2D-CsPbBr_3_ nanoplatelets emit bright blue fluorescence, whereas 1D-CsPbBr_3_ nanowires display intense green emission, both exhibiting strong water resistance and preserved crystallinity, as evidenced by TEM, HRTEM, and SEM analyses. Importantly, antibody-conjugated perovskite nanostructures enable targeted two-photon luminescence (TPL) imaging of specific breast cancer subtypes. The anti-AXL-functionalized 2D-CsPbBr_3_ nanoplatelets selectively illuminate AXL-positive MDA-MB-231 (PR^−^/ER^−^/HER-2^−^) cells, while anti-HER-2 conjugated 1D-CsPbBr_3_ nanowires specifically label HER-2-positive SK-BR-3 cells under two-photon excitation (Figure 14C) [128]. Both systems retain >86% PL intensity after 35 days in water, demonstrating outstanding photostability, selectivity, and biocompatibility.

The major challenges faced by lead halide perovskites in biomedical imaging are their instability in aqueous media, their inherent toxicity due to Pb levels exceeding 200 µg/mL, and their limited scalability. Avugadda et al. [129] demonstrated that silica or polymer encapsulation can preserve PLQYs of ~60% for over two years in water. The toxicity can be reduced by using Pb-free compositions and 2D/3D hybrid structures, which are highly desirable but still require improved stability. Automation of synthesis systems increases the level of scalability, as it generates consistent batches. These are the strategies to facilitate clinical translation of lead halide perovskites in high-sensitivity imaging.

## 5. Challenges and Future Prospective

Although impressive advancements have been made in perovskite crystals, NCs, QDs, and 2D structures, there are other important issues that still restrict their large-scale application and long-term stability. One of them is the case of environmental instability, especially with regard to 3D perovskite crystals and TFs, which are prone to degradation in the presence of moisture, heat, light, and electrical bias. Although PNCs, PQDs, and layered 2DPs provide better stability due to the passivation of the surface and structural confinement, the additional efforts to encapsulation strategies and intrinsic material design are necessary to provide operational stability in the real-world environment.

The other significant threat is the issue of Pb toxicity, which poses both environmental and regulation issues to the optoelectronic and biomedical uses. Considerable attention has been paid to the creation of Pb-free perovskite materials comprising tin, bismuth, antimony, and double-perovskites, but these materials tend to be poorer optoelectronic materials, contain more defects, or have lower long-term stability. Future studies should aim at finding solutions to these shortcomings by using compositional engineering, defect passivation, and new heterostructure architectures that can maintain the desirable optoelectronic performance of lead-based systems and with minimal environmental footprint.

Scalability and reproducibility are also bottlenecks, especially in the case of PNCs, PQDs, and 2DPs prepared by solution-based methods. Further innovation in synthesis, processing, and device integration would be necessary in order to achieve uniform material quality on large scales, surface chemistry at scale, and integrate perovskite nanomaterials in industrial fabrication processes. The technologies that can be used with roll-to-roll manufacturing, blade coating, printing, and low-damage deposition are likely to become more significant in helping to fill the gap between laboratory demonstrations and commercial technologies.

In the future, the rational design of hybrid and mixed-dimensional perovskite architectures (to fabricate bulk films, NCs, QDs, and 2D layers) and capitalize on their respective benefits is one of the most viable directions. With the help of such architectures, charge transport, light emission, environmental stability, and excitonic control can be optimized simultaneously, and novel opportunities of high-performance optoelectronic devices and scalable quantum photonic systems were opened. Further development in this sense, facilitated by in situ characterization, theoretical modeling, and optimization at the device level, would likely quicken the translation of perovskite materials into versatile and efficient technologies.

## 6. Conclusions

Perovskite crystals, PTFs, PNCs, PQDs, and 2DPs, in a continuum of dimensionality, are systems in which optoelectronic behavior is determined by structure-sensitive quantum confinement, surface chemistry, and dielectric environment. In all of them, a remarkable similarity can be observed in their defect-tolerant, electronically determined structures; direct bandgaps, which they can tune; high light–matter interactions; and solution-processing compatibility at low temperature. Such common properties form the basis of the wide use of perovskite materials in LEDs, SCs, PDs, lasers, and new quantum photonic devices.

Meanwhile, definite differences can be found in the functional roles of every structure. Perovskite bulk crystals and PTFs exhibit superior charge transport and scalability, making them the leading ones in large-area photovoltaics and PDs. PNCs and PQDs are regulated by increased quantum confinement with lowering the size and the surface effects, having color-pure emission, ultrafast recombination kinetics, and low-threshold optical gain, being preferable to use in displays, lasers, and quantum sources of light. 2DPs increase one more level of control by using the layered structures, strong excitonic effects, reduction in ion migration, and enhanced environmental stability, which are beneficial to stable light-emitting devices and hybrid heterostructures.

The integration of these material platforms in the future is predicted to take the form of hybrid and hierarchical structures that harness the merits of other dimensionally based structures. The future directions of research should be in scalable fabrication pathways, clean and Pb-free compositions, surface and interface engineering, and the rational combination of mixed-dimensional systems. These approaches will be critical to the transfer of the outstanding optoelectronic capabilities of the perovskite materials that have been demonstrated in the laboratory to those that are strong enough to be commercially viable.

## Figures and Tables

**Table 1:** Tolerance factor and probable crystal structure of perovskites. Reproduced with permission from reference [24]. Copyright 2015, *The Royal Society of Chemistry*.

S. No	Tolerance Factor (*t_f_*)	Descriptions	Structure
1	0.90–1.0	Both A and B cations have an ideal size	Cubic
2	>1.0	A is larger and B is smaller	Tetragonal or Hexagonal
3	0.70–0.90	A small or B large	Orthorhombic or Rhombohedral
4	<0.7	Both A and B have a similar size	Different structures

**Table 2:** Comparative overview of different fabrication techniques, conditions, precursors, advantages, and key challenges.

Synthesis Method	Key Precursor	Temperature and Time	Advantages	Challenges	Refs.
Hot-injection	PbX_2_, Cs-oleate, OA, OLA, ODE	140–200 °C, (seconds to minutes)	Produce uniform size, high PLQY, tunable emission, fast synthesis	Poor stability, sensitive to air/moisture, and low scalability.	[52,53,54]
Room temperature	MAX + PbX_2_ in DMF/DMSO with OA and OLA (LARP)	RT, (minutes to hours)	Energy-efficient, scalable, simple, color-tunable QDs, high PLQY of 92%	Low thermal stability, slower crystallization, and surface defects.	[55,57]
Sol–gel	Metal alkoxides or salts, chelating agent (e.ge, citric acid)	120–250 °C, (hours to days)	High purity, uniform size, consumes low energy compared to solid state, structural tunability	Time-consuming, hard to scale, and sensitive to moisture.	[58,59,60,61,62,63,64,65,81]
Hydrothermal synthesis	Metal halides + alkalis in aqueous solution	150–240 °C, for 12 to 24 h	Low energy, precise size control, enhanced PLQY, strong crystallinity	Slow, pH-sensitive, and difficult impurity removal.	[66,67,68,69]
Solvothermal synthesis	Metal halides + solvent (e.g., DMF/DMSO	80–240 °C, for 6 to 24 h	High crystallinity, fine size control (<20 nm), reduced surface defects	Long reaction time, toxic solvents, and residual ligands affect charge transport.	[70,71,72,73,74,75]
Kinetically controlled space confinement	Lead halide perovskites + layered substrate	100–150 °C, for minutes to hours	High phase purity, precise thickness control (*n* = 1–6), PLQY > 80%	Difficult to scale, requires precise conditions, and toxic solvent.	[76,77,78,79,80]
Solid-state route	SrO, Al_2_O_3_, Nb_2_O_5_, ethanol, deionized water, propanol	800–1200 °C, 6 to 48 h	Simple, scalable, phase-pure, good crystallinity	High energy consumption, large particle size, low surface area.	[82]
Solution process method	Cs/MA/FA + Pb halides, ZnO/AZO/SnO_2_ NP	100–115 °C, for 5 to 10 min	Low cost, scalable, enables tandem solar cells (PCE of 25%), compatible with spin coating	Surface damage from spin-coating, SnO_2_, shows poor UV protection and crystallinity.	[83,84]

**Table 3:** Performance of PTFs, PNCs, PQDs, 2DP, and their applications.

Compound	Structure	PLQY	Efficiency	Applications	Refs.
FAPbI_3_	PTF	90%	20%	LED	[26]
CH_3_NH_3_PbI_3_	PTF	90%	21.6%	SC	[28]
CsPbX_3_ (X = Cl, Br, I)	PNCs	95%	>20%	Display	[34]
MAPbBr_3_	PNCs	92%	12%	LED	[56]
CsPbX_3_ (X = Cl, Br, I)	PQDs	50–90%	20%	Lasing, PD	[14]
CsPbBr_3_	PQDs	>90%	20% green	Laser, LED	[93]
CsPbI_3_	PNCs	>90%	21.3%	LED	[77]
CsPbCl_3_	PNCs	>90%	24.7%	SC	[94]
(BA)_2_PbI_4_	2DP	94%	93%	PD, SC	[46]
CsPbCl_3_: Mn^+2^	PQDs	88%	22.8%	SC	[95]
(PEA)_2_(-MA)_2_Pb_3_Br_10_	2DP	70%	15.5%	LED	[96]

## Data Availability

No new data were created or analyzed in this study.
